# Chromatin as an oxygen sensor and active player in the hypoxia response

**DOI:** 10.1016/j.cellsig.2011.08.019

**Published:** 2012-01

**Authors:** Andrew Melvin, Sonia Rocha

**Affiliations:** Wellcome Trust Centre for Gene Regulation and Expression, College of Life Sciences, MSI/WTB/JBC Complex, Dow Street, University of Dundee, Dundee, DD1 5EH, Scotland, United Kingdom

**Keywords:** HIF, Hypoxia inducible factor, ARNT, Aryl hydrocarbon nuclear translocator, VHL, von Hippel Lindau, PHD, Prolyl-hydroxylase, FIH, Factor inhibiting HIF, ChIP, Chromatin immunoprecipitation, SWI/SNF, Switch/Sucrose NonFermentable, ISWI, Imitation switch, CHD, chromodomain helicase DNA-binding, NURF, nucleosome remodelling factor, CHRAC, Chromatin remodelling and assembly complex, ACF, ATP-utilising chromatin remodelling and assembly factor, NoRC, nucleolar remodelling complex, RSF, Remodelling and spacing factor, WICH, WSTF–ISWI chromatin remodelling complex, NuRD, nucleosome remodelling and histone deacetylase, SRCAP, SNF2-related CBP activator protein, TRRAP, transformation/transcription domain-associated protein/Tip60, HAT, Histone acetyl transferase, HDAC, Histone deacetylase, LSD1, lysine-specific demethylase-1, JmjC, Jumonji C domain, Hypoxia, Chromatin, HIF, Transcription, Chromatin remodellers, JmjC demethylases

## Abstract

Changes in the availability or demand for oxygen induce dramatic changes at the cellular level. Primarily, activation of a family of oxygen labile transcription factors, Hypoxia Inducible Factor (HIF), initiates a variety of cellular processes required to re-instate oxygen homeostasis. Oxygen is sensed by molecular dioxygenases in cells, such as the prolyl-hydroxylases (PHDs), enzymes which are responsible for the oxygen-dependent regulation of HIF. As HIF is a transcription factor it must bind DNA sequences of its target genes possibly in the context of a complex chromatin structure. How chromatin structure changes in response to hypoxia is currently unknown. However, the identification of a novel class of histone demethylases as true dioxygenases suggests that chromatin can act as an oxygen sensor and plays an active role in the coordination of the cellular response to hypoxia. This review will discuss the current knowledge on how hypoxia engages with different proteins involved in chromatin organisation and dynamics.

## Highlights

► Hypoxia activates transcription. ► Chromatin allows for hypoxia induced transcription. ► Chromatin changes via chromatin remodellers. ► Chromatin changes via histone modifications. ► Histone demethylases are oxygen dependent.

## 1 Introduction

Oxygen is essential for the majority of multicellular organisms. As such, variations in oxygen supply and demand within a given time frame activate a variety of pathways, the ultimate aim of which is to re-instate oxygen homeostasis. This is true at the organism level but also true at the cellular level. Oxygen is required for efficient ATP production via oxidative phosphorylation in the mitochrondria, whilst ATP production via glycolysis does not require oxygen, it is much less efficient.

Hypoxia is an important stimulus for physiological processes such as development and adaptation to high altitude living, but it is also an important factor in the pathology of many human diseases [1,2]. These include cancer, diabetes, ageing, and stroke/ischaemia [1,2]. Furthermore, it plays a role in the resistance to therapeutic approaches such as radiotherapy [1,2].

Whilst the understanding of how whole organisms respond to variations in oxygen availability has been greatly enhanced over the last century, with physiology studies [3], the molecular understanding of how oxygen is sensed at the cellular level is much more recent, with the findings made thus far likely being the tip of the iceberg.

The research into oxygen sensing at the cellular level, was greatly enhanced with the discovery of a family of transcription factors that respond to hypoxia, called Hypoxia Inducible Factors (HIF) [1]. HIF is a heterodimer of an oxygen labile subunit, HIF-α, and an oxygen-insensitive HIF-1β, also known as aryl hydrocarbon nuclear translocator (ARNT).

**Fig. 1:**
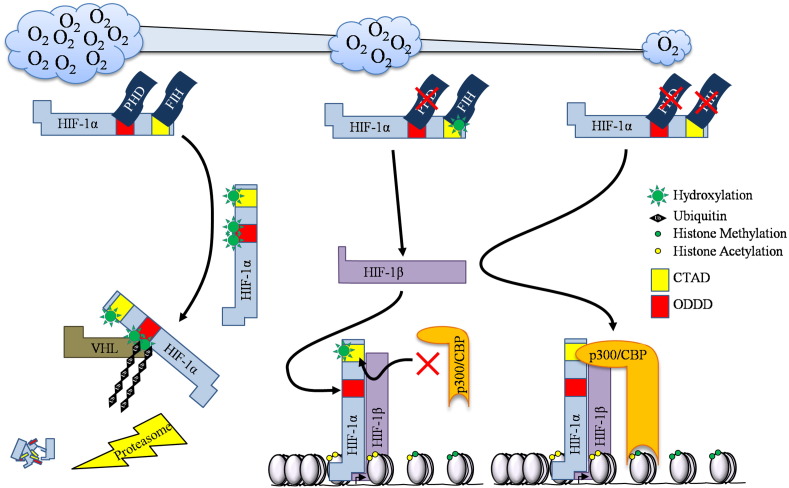
The HIF degradation pathway. In normoxia the hydroxylases (PHDs and FIH) use O_2_ to hydroxylate HIF-1α in the Oxygen Dependent Degradation Domain (ODDD) and the C-Terminal Activation Domain (CTAD). Hydroxylation in the ODDD targets HIF-1α for ubiquitination by the VHL containing E3 ligase complex and HIF-1α is then degraded by the proteasome. In moderate hypoxia the PHDs are inhibited causing HIF-1α accumulation and its dimerisation with HIF-1β. Further decreases in oxygen cause FIH inhibition and subsequent interaction of the HIF-1α-CTAD with co-activators such as p300/CBP.

The tumour suppressor von Hippel Lindau (VHL), as part of the E3 ubiquitin ligase complex, targets HIF-α in the presence of oxygen to be degraded by the proteasome. VHL recognises HIF-α mostly in normoxia, through interaction with hydroxylated proline residues within the oxygen-dependent degradation domain of HIF-α (Fig. 1). Biochemical studies demonstrated that VHL has a 1000 fold increased affinity for hydroxylated HIF, compared to non-hydroxylated [4]. This specific modification of prolines, is mediated by a class of dioxygenases, called Prolyl-Hydroxylases (PHDs). There are 3 PHDs that have demonstrated effects on HIF, PHD1-3 and these enzymes require molecular oxygen for their activity. Another dioxygenase with known effects on HIF is the Factor Inhibiting HIF (FIH). FIH mediates the hydroxylation of asparagine residues within the C-terminus transactivation domains of HIF-α, preventing binding to co-activators such as p300 or CBP [5], and thus limiting HIF transcriptional activity (Fig. 1).

**Fig. 2:**
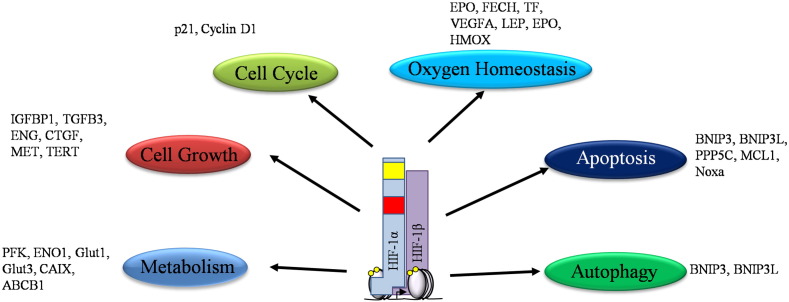
HIF induced transcriptional target and cellular pathways. HIF transcriptional targets are very diverse, involving many aspects of cellular regulation. It is the differential regulation of these HIF target genes that allows for the diverse role of HIF, as well as, the integration of a wide variety of other cellular signals.

HIF can activate many genes involved in many important cellular processes such as cell cycle and cell growth, metabolism, oxygen homeostasis, apoptosis and autophagy (Fig. 2). In fact, recent studies using genomic chromatin immunoprecipitation (ChIP) techniques, ChIP-on-ChIP and ChIP-Sequencing, have demonstrated hundreds of genomic loci, where HIF binds [6,7], suggesting that many genes are under the direct control of these transcription factors [6,7].

The importance of the HIF pathway has been extensively demonstrated by genetic studies: HIF-1β null mice are embryonic lethal with severe defects in many organs [8,9]. Furthermore, conditional HIF-1β knockouts have been made in T-cells [10], β-cells [11] and skin [12], and all of these tissues and cells have several defects. HIF-1α null mice are also embryonic lethal, with defects in heart, brain, vasculature and bone [13–15]. In addition, conditional deletion of HIF-1α has been achieved in macrophages and neutrophils [16], neural cells [17,18], keratinocytes [19], the colon [20] and the liver [21], to name a few, and has been shown to be required for proper function of these tissues. HIF-2α deleted mice present phenotypes that are strain specific [22–24]. However, they all have severe defects in development.

All the PHDs have been deleted in mice, but only PHD2 is embryonic lethal with placental defects [25]. PHD2 also regulates the vascular system in adult mice [26,27]. PHD1 null mice are apparently normal, but demonstrate increased muscle fatigue [28], protection against ischemic/reperfusion injury to the liver [29], and against colitis [30]. PHD3 null mice are born but have defective sympathoadrenal development and are systemically hypotensive [31]. More recently, FIH was deleted in mice, and these were viable, with no apparent developmental defects. Interestingly, FIH null mice have alterations in their metabolism, presenting lower body weight, increased response to insulin and importantly, protection against diet induced weight gain [32]. These results suggest that FIH does not play a role in the control of HIF in developmental hypoxia but perhaps only in the case of pathological hypoxia.

## 2 Chromatin structure

As mentioned before, HIF is an important transcription factor, and as such it requires binding to DNA target sequences in the context of chromatin. Chromatin is a dynamic and complex structure composed of DNA and many proteins. The basic unit of chromatin is the nucleosome. The nucleosome consists of 147 bp of DNA wrapped around an octomer of histones (2 copies of each of the core histones: H2A, H2B, H3 and H4) [33,34]. Nucleosomes are linked with stretches of linker DNA, which incorporate linker histones such as H1 [34]. Nucleosome arrays are further compacted into higher order of chromatin, however detection and analysis methods for these higher order chromatin structures are still not routinely available. In addition, there is no detailed information on how chromatin structure changes in hypoxia.

There are two types of chromatin recognised according to its compaction: heterochromatin and euchromatin. Heterochromatin, is characterised by high levels of compaction and usually associated with silent genes. On the other hand, euchromatin is characterised by lower compaction and associated with actively transcribed genes [35]. A recent biochemical analysis in the model organism Drosophila has identified as many as 5 types of chromatin, two types of active chromatin and 3 types of repressive chromatin [36]. Whether these are present or not in mammalian cells, would require further investigation.

Changes in chromatin are essential for the majority of cellular processes that require access to DNA. These include transcription, DNA replication and DNA repair. In order to access chromatin, organisms have evolved a number of mechanisms whereby DNA-protein contacts are relaxed or tightened depending on the requirement. These mechanisms can be divided in to three broad categories: ATP-dependent chromatin remodelling, post-translation modification of histones and incorporation of histone variants [37–39].

## 3 ATP-dependent chromatin remodellers in hypoxia

**Fig. 3:**
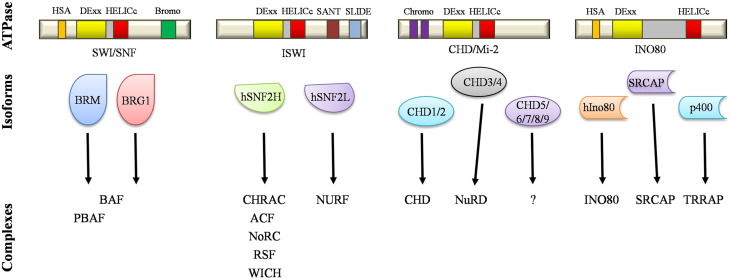
Human ATP dependent chromatin remodellers. The identity of chromatin remodelling complexes is defined by the ATPase subunit, which are in turn defined by their unique structure. All the ATPases shown here contain an ATPase domain (DExx and HELICc with an insertion between them (grey)). Switch/Sucrose non fermentable (SWI/SNF) are characterised by an acetylated histone binding bromodomain. ISWI (Imitation Switch) contain SANT/SLIDE domains. CHD/Mi-2 (chromodomain helicase DNA-binding) contains tandem chromodomains that bind methylated lysine 4 in the histone H3 tail. INO80 are characterised by a larger insertion between the DExx and the HELICc regions. Helicase-SANT domains (HSA) are present in SWI/SNF and INO80. In humans there are multiple isoforms of each SNF2 family member that belong to an even more diverse set of ATPase complexes.

Cells have evolved a variety of enzymatic complexes that utilise the energy of ATP to alter DNA-protein contacts, and hence chromatin structure, according to their needs. These are called ATP-dependent chromatin remodellers, and can change protein-DNA contacts to move or remove nucleosomes. Based on homology and biochemical properties, these enzymes can be divided into a variety of subtypes but the most well characterised are: SWI/SNF (Switch/Sucrose NonFermentable), ISWI (Imitation Switch), CHD/Mi-2 (chromodomain helicase DNA-binding), and INO80 [40] (Fig. 3).

### 3.1 SWI/SNF

**Fig. 4:**
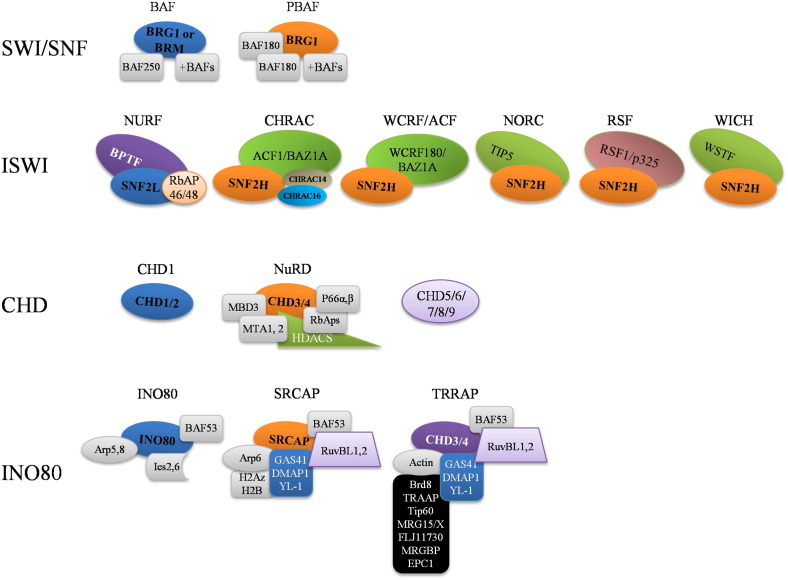
Human ATPase Complexes and their composition. The ATPase subunits SWI/SNF, ISWI, CHD and INO80 form the catalytic centre to a wide variety of chromatin remodelling complexes. The association of the non-catalytic subunits is thought to drive the specificity of the chromatin remodelling complexes. The catalytic subunit is indicated in bold.

Perhaps the most studied family of chromatin remodellers, SWI/SNF, is an evolutionary conserved multi-subunit complex (Fig. 4). It possesses one of two possible catalytic subunits, BRG1 or BRM, and a variety of accessory subunits that confer DNA binding and specificity [41]. It is mostly associated with activation of transcription but it is also necessary to promote transcription repression in certain circumstances [42]. Whilst BRG1 is essential for embryo development [43], BRM is not [44]. In addition, some of its subunits have been found deleted or mutated in a variety of cancers [45], further demonstrating their importance. Despite this, the function of these complexes in vivo, and in particular, in response to a given stimuli is still not well understood. This is also true in the context of hypoxia.

Very little information exists about chromatin structure and its dynamics in hypoxia. However, two independent studies have revealed important functions for SWI/SNF in the hypoxia response [46,47]. Whilst BRM and BRG1 were found at the EPO promoter and demonstrated to be required for HIF-mediated induction of this gene [46]; BRG1 but not BRM was found at the HIF-1α gene itself [47]. Importantly, SWI/SNF was shown to be required not only for full HIF levels following hypoxia, but also required for hypoxia induced cell cycle arrest [47]. The genetic knockout of the mouse homologue of BAF155, SRG3, has been published [48]. The mice show peri-implantation lethality, with SRG3 being required for angiogenesis and visceral endoderm development. The genes deregulated were identified as Angiopoietin1, Tie2 and EphrinB2 [48]. Angiopoetin and Tie-2 are also deregulated in HIF-1β deleted mice [9]. However, the levels of HIF in SRG3 deleted mice have not been investigated. The connection between BRG1 and vascularisation is further supported with genetic studies where conditional deletion of BRG1 was conducted in the hematopoietic and endothelial cells. These mice showed important defects in erythropoiesis and vascular development [49].

These studies demonstrated that chromatin and chromatin remodelling enzymes do play an active role in the cellular response to hypoxia and should be further investigated.

### 3.2 ISWI

ISWI is another important chromatin remodelling complex conserved through evolution [35]. In humans, it is comprised of several different complexes, sharing two possible catalytic subunits, hSNF2H and hSNF2L (Fig. 3). In addition, accessory proteins (Fig. 4) define the different complexes observed: NURF (nucleosome remodelling factor), CHRAC (chromatin accessibility complex), ACF (ATP-dependent chromatin and remodelling factor), NoRC (nucleolar remodelling complex), RSF (Remodelling and Spacing Factor) and WICH (WSTF–ISWI chromatin remodelling complex). hSNF2H is essential for embryo development with defects observed at the peri-implantation stage [50], however, there is no information of the role of hSNF2L in development thus far.

ISWI is involved in a variety of important biological processes [51]. These include DNA replication and repair, regulation of transcription, and regulation of chromosome structure [51]. In in vitro experiments and in yeast, ISWI has been shown to promote even spacing of nucleosomes and as such has been thought as mostly a transcriptional repressor [51]. In addition, one of ISWI's complexes RSF has been shown to promote tumourigenesis and genomic instability [52,53].

ISWI function has not been investigated in the context of a given stimulus thus far, and as such there is no information concerning the involvement of ISWI in the cellular response to hypoxia. Further work is therefore necessary to investigate in more detail how chromatin remodellers such as ISWI are modulated by hypoxia.

### 3.3 CHD/Mi-2

Mammalian CHD chromatin remodellers (Figs. 3, 4) all contain chromodomains, which are involved in chromatin remodelling and binding to methylated lysine residues [54]. Amongst the CHD family members is the NuRD complex (nucleosome remodelling and histone deacetylase) [37]. Despite being conserved in many organisms, CHD function and in particular regulation are not well understood [37]. However, several of the CHD genes are required for embryo development. These include CHD2 and CHD8. Furthermore, some of the CHD genes are found mutated in human diseases such as the CHARGE syndrome (CHD7) [55]. In a recent study analysing gastric and colorectal cancer, all CHD genes were found to be mutated [56]. Furthermore, CHD5 promoter can be hypermethylated in Glioma, Breast and Colon cancers [57]. These studies once again demonstrate the important role of these complexes for normal cellular function.

Whilst there is no direct evidence that CHD complexes play a role in the cellular response to hypoxia, different studies have demonstrated that MTA1 (a member of the NURD complex (Fig. 4)) positively correlates with HIF-1 activity and angiogenesis [58,59]. In addition, MTA1 increases the levels of HIF-induced VEGF, an important HIF target for promoting angiogenesis in tumours [59]. However, whether MTA1 regulation of HIF requires CHD motor activity has not been investigated.

### 3.4 INO80

The INO80 family of chromatin remodellers is composed of three different protein complexes, characterised by having an insertion in their ATPase, creating a split-ATPase domain: INO80, SRCAP (SNF2-related CBP activator protein) and TRRAP (transformation/transcription domain-associated protein)/Tip60 (Fig. 4) [37]. These complexes are involved in transcription regulation and DNA repair [60]. Of the INO80 complexes known, only TRRAP has been reported to be essential for embryo development [61]. Interestingly, two components of all INO80 complexes RuvBL1 (Pontin) and RuvBL2 (Reptin) have been found deregulated in human cancers [62]. These proteins associate with Histone Acetyl Transferases (HATs) and Histone Deacetylases (HDACs) and can regulate the activity of a number of important transcription factors, notibly, c-myc, β-catenin and HIF-1α [62].

Whether the INO80 complexes play an active role in the cellular response to hypoxia has not been formally investigated. However, an interesting study by the Baek group has demonstrated that Reptin is methylated by the methyl transferase Ga9 in hypoxia and that it associates with HIF-1 repressing a subset of HIF-dependent target genes in hypoxia [63]. This suggests that at least Reptin plays a role in the response to hypoxia. This group then demonstrated that as with Reptin, G9a could methylate Pontin in hypoxia but that this activated a different set of HIF-dependent targets in hypoxia [64]. However, there was no evidence that chromatin remodelling was involved in this process, leaving the question open as to whether the motor activity of these complexes is required for any of the responses observed in hypoxia.

## 4 Post-translational histone modifications

One of the fastest ways of changing protein function is through posttranslational modifications. Histone proteins are one of the most conserved proteins known and are themselves targets of a multitude of posttranslational modifications that act to alter contacts with DNA or with other proteins. Histones can be ubiquitinated, sumoylated, phosphorylated, acetylated, methylated, citrullinated and hydroxylated [38]. Whilst some of these modifications have been associated with a given function, many have not. Here we will focus on modifications with known or possible roles in the hypoxia response.

### 4.1 Histone acetylation

Perhaps the best studied histone modification, acetylation is thought to add a charge to the histone, and hence loosen histone contacts with DNA. In addition, acetylated histones create a binding site for specific proteins such as chromatin remodellers that possess a bromodomain [37,65]. Acetylation is a dynamic modification, being placed by HATs and removed by HDACs. For the majority of genes, acetylation of histones correlates with active transcription and a more open chromatin structure, such as the one found in euchromatin. On the other hand, removal of acetylation associates with transcriptional repression and heterochromatin.

Thus far, only one study has reported global changes in histone acetylation in hypoxia [66]. However, this study used prolonged and severe hypoxia conditions for their analysis. Nevertheless, specific genes have been analysed for this modification. For example, hypoxia induces increases in acetylation in HIF target genes such as CA9 and VEGF [67,68]. Furthermore, around 40% of HIF target genes are dependent of HIF's association with the HAT p300 or CBP [69]. In addition to HIF binding to p300/CBP, HIF has been shown to associate with other HATs, for example PCAF and SRC-3 [70–72]. And these have shown to be required for proper HIF transcriptional activation of its target genes.

Interestingly, HIF also binds to HDACs and these are required for proper HIF activity [73]. Whilst some of these effects might be due to de-acetylation of HIF or HIF associated proteins, histone de-acetylation might also play a part. However, further studies are needed at HIF target gene promoters to fully investigate these opposing effects of acetylation.

In addition, sirtuins, which are enzymes with histone deacetylase activity [74], have been shown to be important for HIF activity. Sirt1 was shown to be required for HIF activity [75,76], whilst Sirt3 and Sirt6 inhibit HIF [77–80]. In addition, Sirt1 was shown to be hypoxia inducible [81].

### 4.2 Histone methylation

Unlike acetylation, methylation does not change the histone charge. However it does alter the histone basic and hydrophobic properties, changing its affinity for proteins and creating binding sites for certain specific protein domains such as plant homeodomain or chromodomain [82]. Methylation is added to histones by methyl-transferases, which can be divided into three types: SET domain lysine methyltransferases; non-SET domain lysine transferases and arginine methyltransferases [83]. The addition of these methyl groups can result in both transcriptional activation and repression.

Only three of the known histone methyltransferases have been studied in hypoxia, G9a, Suv39h, and PRMT2 [63,64,84–86]. Hypoxia has been shown to increase G9a activity and levels [63,85]. Whilst G9a was shown to be responsible for increased di-methylated H3K9 [85], increased G9a activity also induced methylation of non-histone proteins [63], and this was responsible for specific HIF target gene repression. Suv39h1 and Suv39h2 were recently shown to be important methyltransferases that are induced by hypoxia, with critical roles in embryo development [86]. In addition, PRMT2 was increased in mice exposed to hypoxia [84]. Despite these interesting findings, there is no information on the other histone methyl-transferases or any global changes in their activity. Furthermore, their functional importance has only been assessed in certain cases and further studies are needed to firmly conclude the contribution of these enzymes towards the cellular response to hypoxia.

Unlike acetylation, for many years, methylation was thought to be an irreversible modification. The first histone demethylase that was identified is the lysine-specific demethylase-1 (LSD1) [87]. Since then this area of research has grown exponentially with the identification of the Jumonji C (JmjC) domain containing demethylases. Importantly, in the context of this review, the JmjC containing enzymes are α-ketoglutarate, Fe^2+^, dioxygenases, with a structure very similar to that of FIH [88]. This indicates that this class of demethylases are perfectly poised to respond to hypoxia.

Only a few studies have investigated changes in histone methylation after hypoxia. In addition, these studies have been done in different cellular systems, different times of hypoxia exposure and different O_2_ concentrations. In the first study, where histone methylation was analysed at global levels, hypoxia was performed using nearly anoxic levels (0.2% O_2_), and histone marks were analysed 48 h later. This study revealed dramatic changes in both transcription and histone marks, with both active and repressive marks being induced [66]. These results suggest either a global increase in histone methyltransferase activity or a decrease in histone demethylase function. An additional study, used mouse macrophage cells to investigate changes in global and localised histone marks in inflammatory genes following 24 h of exposure to decreasing levels of oxygen [89]. This study found that changes in histone methylation were visible only below 3% O_2_. It was found that H3K9 di- and tri-methylation were increased and also H3K36 tri-methylation was globally increased in macrophages following 24 hours exposure to 1% O_2_ [89]. The authors suggest that this was due to histone demethylase inhibition at this oxygen concentration. A different study, investigated H3K4 tri-methylation levels following hypoxia (1% O_2_) both globally and locally [90]. Hypoxia induced increases in this modification in all the cell lines analysed. The authors went on to show, that this increase was due to inhibition of histone demethylase activity in hypoxia [90]. These exciting new findings will pave the way for further studies investigating global and localised histone methylation changes. The combination of population and single cell analyses should provide additional information on how hypoxia modulates the chromatin landscape. The identification of JmjC demethylases suggests that chromatin will react very rapidly to changes in oxygen but also to iron and metabolite availability.

## 5 JmjC demethylases and hypoxia

There are over 100 proteins containing JmjC domains so far identified in different organisms [91]. In humans 30 such proteins have been identified [92]. These enzymes can remove methyl groups from lysines and arginine residues on histones and other proteins [91,92]. Extensive studies in vitro and some studies in vivo, have revealed some specificity for the JmjC demethylase (Table 1). Given their enzymatic requirements for molecular oxygen, these proteins are placed as perfect oxygen sensors, giving rise to the notion that chromatin could act as an oxygen responsive structure. Moreover, some of these enzymes are direct targets of the HIF transcription factor [93–97], further suggesting their functional involvement in the response to hypoxia. In addition, the majority of the JmjC demethylases are hypoxia inducible (Table 1).

The functional significance of these enzymes towards the response to hypoxia is only now being analysed, and given the great number of enzymes, this task will be laborious. However, some evidence of their significance has already been demonstrated. For example, JMJD3 (KDM6B) knockout mice are lethal, indicating that this enzyme is required for proper embryonic development [98]. Similarly, JMJD6, Jarid2, JMJD2B (KDM4B), FBXL-10 (KDM2B), Hairless, JMJD1A (KDM3A) all have reported defects in a variety of organs and cellular processes [92]. PHF8 and Hairless are found mutated in human diseases [99,100]. In addition, a systematic sequencing approach in human renal cancers has identified UTX (KDM6A), Jarid1C (KDM5C) as genes mutated in renal carcinoma [101,102].

JmjC specific action in hypoxia has been analysed in only a few studies so far, with JMJD1A being the one mostly studied [94–97]. JMJD1A was found to be required for hypoxia induction of ADM and GDF15 in renal and colon cancer cells [97]. In a separate study, Zhou and colleagues demonstrated that hypoxia inhibits JARID1A (KDM5A) to increase the levels of H3K4 tri-methylation [90]. Furthermore, this was increased for hypoxia inducible targets such as HOMX1 and DAF but not others [90]. However, no additional study has combined siRNA, deletion or overexpression of other JmjC proteins with hypoxia to determine their relevance in a given cell system. Given their documented importance in development and human disease, further studies are needed to determine the relative and cell type contribution of these enzymes to chromatin organisation, transcription rates and the cellular response to hypoxia.

## 6 Histone variants

As mentioned before, the basic unit of chromatin is the nucleosome, which is composed of two copies of each of the canonical histones H3, H4, H2A and H2B. An additional mechanism to alter chromatin structure and function is the replacement of canonical histones with histone variants [39]. Amongst the canonical histones, only H4 has no variant identified. Some histone variants only change by a few residues, whilst other have considerable differences both at the sequence and structural levels. Table 2 represents the known histone variants, and their described roles and phenotypes. For an extensive review please see [103,104].

Histone variants can mark areas of DNA damage such as H2Ax, or important for cell division, for example CENPA [39]. In addition, genetic studies have documented the importance of histone variants for development [104]. In the context of hypoxia, only one variant has been analysed and described, H2Ax [105,106]. In fact, a genetic study has indicated that H2Ax is required for hypoxia induced neo-vascularisation [105]. In addition, phosphorylated H2Ax has been reported in many studies as a consequence of hypoxia induced ATR activity [106]. Whether some other histone variants are deposited in hypoxia or even play a role in the hypoxia response has not been investigated.

## 7 DNA methylation

An additional mechanism to change chromatin and transcription is by methylation of DNA itself. Generally, DNA methylation occurs at CpG nucleotides, which are found in repetitive sequences in genes but also with promoters of genes [107]. This modification is catalysed by DNA methyltransferases [108]. Like histone methylation, DNA methylation is also dynamic, although the exact mechanism of DNA demethylation is unknown, Basic Excision Repair (BER) enzymes have been associated with the process of active DNA demethylation [108].

Promoter hyper-methylation is associated with transcriptional silencing, as the methylation prevents the binding of the majority of transcription factors thus far investigated [107]. In addition, recent studies have demonstrated that DNA methylation inhibits the recruitment of additional factors involved in transcriptional regulation such as histone H3 K36 demethylase, KDM2A [109,110]. These studies demonstrated an intrinsic connection between DNA and histone methylation, to help orchestrate proper chromatin structure.

With regard with hypoxia, many studies have demonstrated that promoter hypermethylation prevents HIF binding to its targets. Such examples are: BNIP3, CA9, and PHD3 [111–115]. Globally hypoxia has been shown to prevent DNA methylation by inhibition of the expression of DNMT [116,117] and also by induction of MAT2A [118]. However, exposure to chronic hypoxia in animal models has been linked to increased DNA methylation profiles [119]. Again temporal and cell type specific analyses will be required to properly define the role of hypoxia in the control of DNA methylation. In addition, given the crosstalk between DNA and histone methylation, it will be very interesting to investigate how hypoxia regulates both pathways to obtain the proper cellular response.

## 8 Summary

**Fig. 5:**
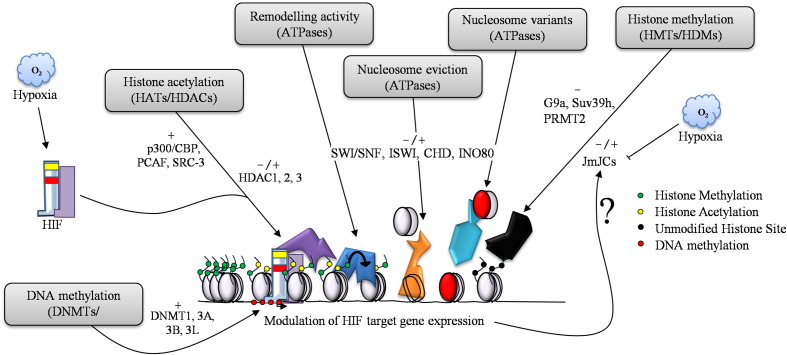
Hypoxia and chromatin crosstalk. Many aspects of chromatin regulation are involved in the modulation of HIF target genes in hypoxia. HIF recruits co-activators such as p300/CBP and depends on histone de-acetylases HDACs for activation, as well as, repression. ATP dependent chromatin remodellers such as SWI/SNF are involved in the regulation of HIF and HIF target genes, however, very little information exists for the roles of the other chromatin remodellers and to what extent HIF may interact/recruit them directly. Histone methylation is an emerging area of hypoxia research as the JmjC de-methylases depend on oxygen for their activity. Furthermore, the majority of the 28 human JmjC proteins are transcriptionally up-regulated by HIF. (−/+) indicates the known effects on transcription.

This review has summarised the crosstalk between hypoxia and chromatin (Fig. 5). Chromatin could act as a primary oxygen sensor, with changes in histone and protein methylation giving rise to further structural changes in chromatin. As most of the hypoxia responses rely on transcription, chromatin must accommodate rapid changes in ATP supply and coordinate correct access of transcription factors to the DNA sequences of its targets. Whilst chromatin structure has not been studied in hypoxia thus far, global changes to histones have been detected. Furthermore, the identification of JmjC domain histone demethylases as oxygen dependent enzymes, further supports the idea that chromatin will sense oxygen changes rapidly (Fig. 5). Exciting future work in these areas will most certainly reveal new mechanisms by which hypoxia changes the cell's signalling pathways.

## Figures and Tables

**Table 1:** Name and function of human JmjC proteins and their connection with hypoxia. Gene names are given in bold. Fold induction shown from microarray experiments conducted in U2OS cells (Melvin and Rocha, unpublished observations).

Human JmjC proteins	Site specificity	Hypoxia inducible	Function	References
**KDM2A**/JHDM1A/FBXL-ll	H3K36me1;H3K4me3	Y [120]	Enhances DNA repair by nonhomologous end-joining. [121]	
			Regulator of rRNA transcription in response to starvation. [122]	
**KDM2B**/JHDM1B/FBXL-l0	H3K36me1/2	Y [120]	Potential tumour suppressor [123]	
		1.43 fold	Regulates cell proliferation and senescence through p15(Ink4b). [124]	
**JHDM1D**/KIAA1718		Y [120]		
		Y [125]		
		1.98 fold		
**PHF8**	H3K9me1/2	Y [120]	But it has preferential binding of H3K4me3	[127]
			Associated with cleft lip/palate and mental retardation [126]	
**PHF2**	H3K9Me2	Maybe [120]	Activated through PKA-mediated by phosphorylation	[128]
**JMJD8**/LOC339123				
**KDM3B**/JMJDlB/JHDM2B	H3K9me1/2	Y [120]		
		Y [125]		
**KDM3A**/JMJD1A/JHDM2A	H3K9me1/2	Y [125]	Metabloic gene expression and weight control [130,131]	Identified by [132]
		Y [96]		Array with KD — [133]
		Y [129]		
		Y [89]		
		1.7 fold		
**JMJD1C**		Y [120]		
		− 1.54 and + 1.34 fold?		
**Hairless**				
**JMJD4**				
**JMJD6**/PTDSR	H3R2me2;H4R3me2	Y [120]	Catalyses Lysyl-Hydroxylation of U2AF65, a Protein Associated with RNA Splicing [134]	
		Y [125]		
		1.6 fold		
**HSPBAPl**				
**HIFAN**/FIHl	HIF-1α	No		
**KDM4C**/JMJD2C/GASC1	H3K9/K36me2/3	Y [120]	+ In oesophageal sq. Carcinoma [135]	
		Y [125]	RNAi reduced proliferation [136,137]	
		1.8 fold		
**KDM4A**/JMJD2A/JHDM3	H3K9/K36me2/3			[138]
**KDM4B**/JMJD2B	H3K9me2/3	Y [120]	Coordinates H3K4/H3K9 methylation and promotes hormonally responsive breast carcinogenesis. [139]	[139,140]
		Y [96]		
		Y [89]		
		1.5 fold		
**KDM4D**/JMJD2D	H3K9me2/3	Y [89]	Only up in RAW264.7macrophages not peritoneal macrophages	[89]
**KDM5D**/SMCY/JARID1D	H3K4me2/3	Y [120]		
**KDM5C**/SMCX/JARID1C	H3K4me2/3	Y [120]		
		Y [125]		
**KDM5B**/PLU-1/JARID1B	H3K4me2/3	Y [120]	+ In breast and testis cancer [141,142]	
		Y [125]	RNAi reduces proliferation [143]	
		1.45 fold		
**KDM5A**/RBP-2/JARID1A	H3K4me2/3	Y [120]		[90]
		Y [125]		
		N [90] in BEAS-2B		
**KDM6A**/UTX	H3K27me2/3	Y [120]		
**UTY**	H3K27me2/3			
**KDM6B**/JMJD3	H3K27me2/3	Y [120]		
		1.57 fold		
**JARID2**		Y [120]		
		1.33 fold		
**JMJD7**/PLA2G4-B		− 1.68 fold		
**JMJD5**			Potential tumour suppressor [123]	

**Table 2:** Histone variants identified so far, their localisation and known genetic phenotype (information adapted from [104]).

Variant	Family	Location	Phenotype
H3.3	H3	Euchromatin, gene bodies, promoters	
H3T	H3	Testes	
CENP-A	H3	Centromere	Embryonic lethal
H2A.Z	H2A	Regulatory elements, promoter, pericentric repeats	Embryonic lethal
H2A.X	H2A	XY Body, sites of double strand DNA breaks	Male sterility, reduced fecundity in females
macroH2A	H2A	Inactive X, promoters	Severe brain malformations (zebrafish)
H2AL	H2A	Percentric repeats	
H2A.Bbd	H2A		
TH2A		Testes	
TH2B	H2B	Testes	
